# Glucosyltransferase Activity of *Clostridium difficile* Toxin B Triggers Autophagy-mediated Cell Growth Arrest

**DOI:** 10.1038/s41598-017-11336-4

**Published:** 2017-09-05

**Authors:** Ruina He, Jingyu Peng, Pengfei Yuan, Junjiao Yang, Xiaoji Wu, Yinan Wang, Wensheng Wei

**Affiliations:** Biomedical Institute for Pioneering Investigation via Convergence (BIOPIC), Beijing Advanced Innovation Center for Genomics, Peking-Tsinghua Center for Life Sciences, State Key Laboratory of Protein and Plant Gene Research, School of Life Sciences, Peking University, Beijing, 100871 China

## Abstract

Autophagy is a bulk cell-degradation process that occurs through the lysosomal machinery, and many reports have shown that it participates in microbial pathogenicity. However, the role of autophagy in *Clostridium difficile* infection (CDI), the leading cause of antibiotics-associated diarrhea, pseudomembranous colitis and even death in severe cases, is not clear. Here we report that the major virulent factor toxin B (TcdB) of *Clostridium difficile* elicits a strong autophagy response in host cells through its glucosyltransferase activity. Using a variety of autophagy-deficient cell lines, i.e. HeLa/*ATG7* ^−/−^, MEF/*atg7* ^−/−^, MEF/*tsc2* ^−/−^, we demonstrate that toxin-triggered autophagy inhibits host cell proliferation, which contributes to TcdB-caused cytopathic biological effects. We further show that both the PI3K complex and mTOR pathway play important roles in this autophagy induction process and consequent cytopathic event. Although the glucosyltransferase activity of TcdB is responsible for inducing both cell rounding and autophagy, there is no evidence suggesting the causal relationship between these two events. Taken together, our data demonstrate for the first time that the glucosyltransferase enzymatic activity of a pathogenic bacteria is responsible for host autophagy induction and the following cell growth arrest, providing a new paradigm for the role of autophagy in host defense mechanisms upon pathogenic infection.

## Introduction

As an evolutionarily conserved process, autophagy plays essential roles in cell survival, development, and homeostasis by mediating the bulk degradation and recycling of cellular macromolecules and organelles^1, 2^. During this “self-consuming” process, an isolated membrane called phagophore forms and extends around its substrates in the cytoplasm. This closed double-membrane vacuole, called autophagosome, fuses with lysosomes to form autolysosome and degrades the contents within the lumen^1–3^. Autophagy is regulated through different signaling pathways, including the mammalian target of rapamycin (mTOR)-related signaling acting as a master negative regulator^4, 5^. TSC2 (tuberous sclerosis complex), a negative upstream effector, receives signals from AMPK which directly senses the cellular energy level, to inhibit mTOR signaling, leading to the activation of autophagy response^6, 7^. As for the molecular machinery, the initiation of autophagy requires the class III phosphatidylinositol 3-kinase (PI3K) complex, which is mainly composed of VPS34, Beclin 1 and ATG14L or UVRAG^8^. Two ubiquitin-like systems are essential in the subsequent autophagosome generation, in which ATG12 is covalently linked to ATG5 for the elongation of the isolation membrane^9^, and LC3 is conjugated to phosphatidylethanolamine^10^. ATG7 is an E1-like ubiquitin enzyme that plays a crucial role in both conjugation systems^11^.

Autophagy is involved in a wide range of cancers, neurodegenerative and infectious diseases^12, 13^. There is growing evidence showing that autophagy also plays a role in cell proliferation. The proteasome inhibitor-induced autophagy has been shown to be responsible for proliferative arrest in colon cancer cells^14^, and the experimental mouse model with deletion of some autophagy proteins, Beclin 1 and Ambra 1, showed an increase in cell proliferation^15, 16^. Recently, various pathogens have been shown to interfere with this pathway^17–21^. For instance, autophagy is important for toxin-induced cell lysis, in a format characteristic of programmed cell death, including toxins such as ricin, Shiga toxin, and diphtheria toxin^22–24^.

*Clostridium difficile* (*C*. *difficile*) infections (CDIs) are the major cause of antibiotic-associated pseudomembranous colitis, and lead to severe diarrhea, ruptured colon, perforated bowels, kidney failure and death^25–27^. There has been a dramatic increase in CDIs over the past decade, largely due to the excessive use of antibiotics and the emergence of more virulent strains, such as strain B-1470. *C*. *difficile* has become the leading cause of healthcare-associated infections^27, 28^. As a gram-positive anaerobe bacterium, *C*. *difficile* exerts its pathogenic effects mainly by producing two virulent factors, enterotoxin A (TcdA) and cytotoxin B (TcdB)^29–31^. Both toxins enter cells via receptor-mediated endocytosis^32–34^. Their glucosyltransferase (GT) domains are subsequently released into the cytoplasm where they mono-glucosylate small GTPases of the Rho subfamily^35, 36^, such as RhoA, Rac1, Cdc42, and TC10, by using the UDP-glucoses as co-substrates^37–44^. These reactions lead to actin condensation and consequently cell-rounding, membrane blebbing, and eventually cell death^45–50^. While both toxins are glucosyltransferases with similar structures that act on a variety of cell types, TcdB exhibits a 100-fold higher rate of enzymatic activity than TcdA^51, 52^. A mutant study in a hamster disease model provided evidence that TcdB, but not TcdA, was essential for virulence^53^. However, another study suggested that both toxins were needed for the virulence of *C*. *difficile* ^54^. Despite this controversy, the role of TcdB in CDIs is indispensable. The cytotoxin of TcdB elicits its biological effects by inhibiting cell proliferation^55^ and even inducing both apoptotic response^56, 57^ and necrotic cell death^58^ in a variety of human cells. It remains to be understood how exactly TcdB exerts its cytopathic and cytotoxic effects.

In this study, we provide evidence that host autophagy, triggered by TcdB from *C*. *difficile* through its glucosyltransferase activity, is critical for TcdB to inhibit host cell proliferation which plays as an important role in the biologic effects of TcdB^55^.

## Results

### TcdB Triggers Autophagy Induction in Host Cells

**Figure 1:**
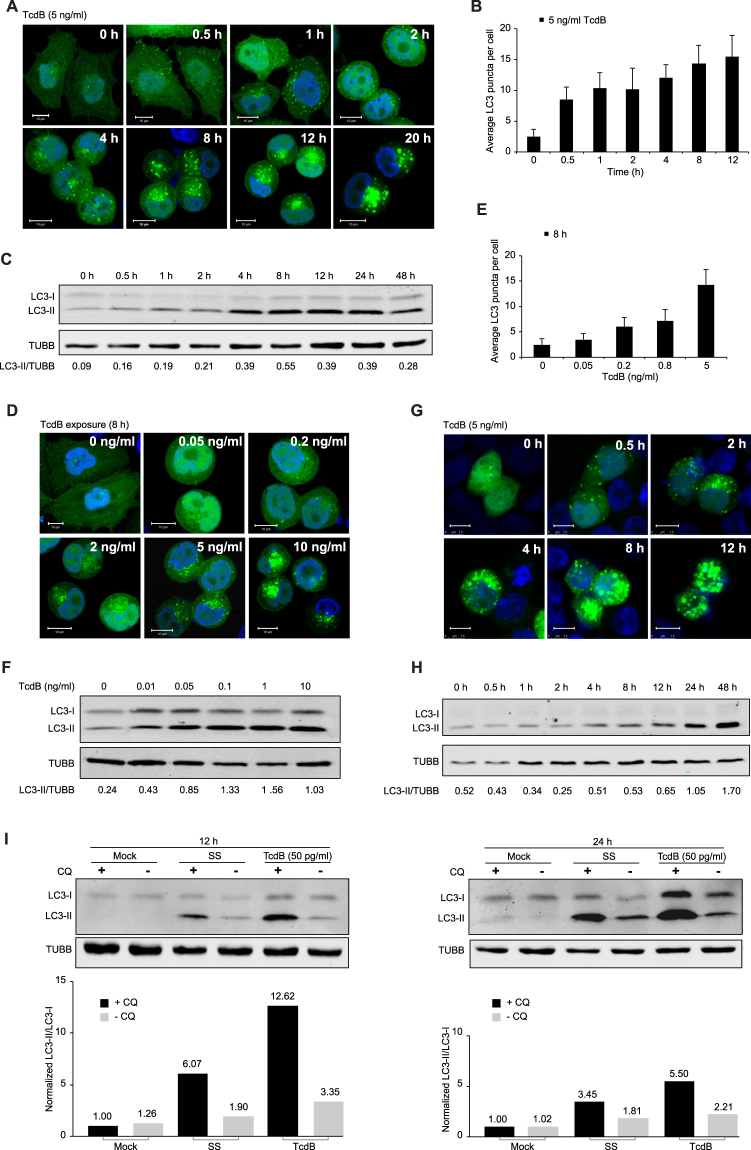
TcdB triggers autophagosome accumulation in host cells. (**A**) Autophagosome accumulation with TcdB treatment in HeLa cells. Cells stably expressing GFP-LC3 were treated by 5 ng/ml of TcdB and analyzed for GFP-LC3-positive autophagosome signals at different time points by fluorescence microscopy. DAPI was used to stain nuclear DNA in this and other figures. Scale bar = 10 μm. Representative images of three independent experiments were shown. (**B**) The statistic results of (**A**). For each time point, the average number of LC3 puncta per cell were counted from over 50 cells randomly. (**C**) Cells were analyzed by immunoblotting on the levels of endogenous LC3-I and LC3-II with the treatment of TcdB (5 ng/ml) under different time points. β-tubulin bands were measured as internal controls for this and other figures. All bands were calculated by Image J. (Full-length blots are presented in Supplementary Fig. S9). (**D**) Autophagosome accumulation with increasing dosage of TcdB in HeLa cells. Cells stably expressing GFP-LC3 were treated by various amount of TcdB for 8 h and analyzed for GFP-LC3-positive autophagosome signals by fluorescence microscopy. Scale bar = 10 μm. Representative images of three independent experiments were shown. (**E**) The statistic results of (**D**). For each time point, the average number of LC3 puncta per cell were counted from over 50 cells. (**F**) Cells were analyzed by immunoblotting on the levels of endogenous LC3-I and LC3-II with the treatment of TcdB of different concentration for 8 h. All bands were calculated by Image J for this and other figures. (**G**) Autophagosome accumulation with TcdB treatment in HT-29 cells. Cells transiently transfected with GFP-LC3 were treated by 5 ng/ml of TcdB and analyzed for GFP-LC3-positive autophagosome signals. Scale bar = 7.5 μm. (**H**) Cells were analyzed by immunoblotting on the levels of endogenous LC3-I and LC3-II with the treatment of TcdB (5 ng/ml) under different time points. (Full-length blots are presented in Supplementary Fig. S10) (**I**). Assay of TcdB-triggered autophagy flux. HeLa cells were treated by 50 pg/ml of TcdB, or serum starvation (SS) for 12 or 24 h, with or without the lysosomal inhibitor chloroquine (CQ, 7 μM), and cell lysates were analyzed by immunoblotting for levels of endogenous LC3-I and LC3-II (Top). Each band was normalized with mock control treated by CQ. (Full-length blots are presented in Supplementary Fig. S11).

To investigate the role of host autophagy in *C*. *difficile* toxin B (TcdB) infection process, we first set out to determine whether and how TcdB affects the cellular autophagy level. By assessing the dynamics of LC3 as indicated by the appearance of the autophagosome-specific marker lipidated LC3 (LC3-II) converted from its unconjugated form (LC3-I)^59, 60^, we could monitor the autophagy activity over the course of toxin exposure. HeLa cells stably expressing GFP-LC3 were incubated with TcdB of various concentrations over different time periods. Aside from the expected cell-rounding phenotype, TcdB-intoxicated cells showed an increase in the number of autophagosomes (Fig. 1A). The statistical average number of LC3 puncta in each cell further confirmed that the accumulation of autophagosomes correlated positively with toxin-exposure time at a fixed TcdB dose (5 ng/ml) (Fig. 1B). The immunoblotting analysis showed more LC3-II accumulated with longer toxin-exposure time (Fig. 1C), which also indicated the increase of autophagosomes by TcdB. Moreover, the increase of autophagosomes correlated with the amount of toxin when the exposure time was fixed (8 h) (Fig. 1D). Statistically, it showed clearly that the average number of LC3 puncta in each cell increased with the amount of toxin added (Fig. 1E). Consistently, more LC3-II accumulated under higher dosage of TcdB, shown in the immunoblotting assay (Fig. 1F). Interestingly, cells were sensitive to TcdB exposure such that as low as 0.5 pg/ml of toxin was sufficient to induce autophagosome formation (Supplementary Fig. S1A). We also found that TcdA, another key virulent factor of *C*. *difficile* ^29–31^, leads to autophagosome formation and induces autophagy in HeLa cells (Supplementary Fig. S1B,C). In agreement with the results from HeLa cells, TcdB also triggered autophagosome accumulation in the human intestinal cells HT-29 (Fig. 1G and H), the natural host targets of TcdB.

Considering that autophagy is a dynamic process, and autophagosomes are intermediate structures, their accumulation could result from either *de novo* induction of autophagy or inhibition of autophagosome degradation. In order to monitor the autophagy flux under TcdB treatment, we used the lysosomal inhibitor, chloroquine (CQ), to block autophagosome degradation^60, 61^. The accumulation of LC3-II triggered by TcdB was significantly enhanced in the presence of CQ for both 12 and 24 h toxin exposure (Fig. 1I), similar to the effects of the serum starvation (SS) treatment, the physiological inducer of autophagy. The quantification results further showed that the turnover rate of LC3-I to LC3-II with CQ is almost 4 times of that without CQ under TcdB treatment, which is greatly higher than the mock control and SS treatment (Fig. 1I). These data indicated that TcdB indeed increased the autophagy flux. In fact, the TcdB-triggered Rac1 glycosylation was delayed by 0.5 h with the addition of CQ, suggesting that CQ slightly inhibits the endocytosis of TcdB (Supplementary Fig. S2). It rules out the possibility that CQ helps the endocytosis of TcdB to promote the autophagy response. Altogether, these results suggested that the autophagosome accumulation results mainly from the TcdB-mediated induction of autophagy rather than its inhibition of autophagosome degradation.

### Autophagy Induction Facilitates TcdB-Caused Cell Proliferation Inhibition

**Figure 2:**
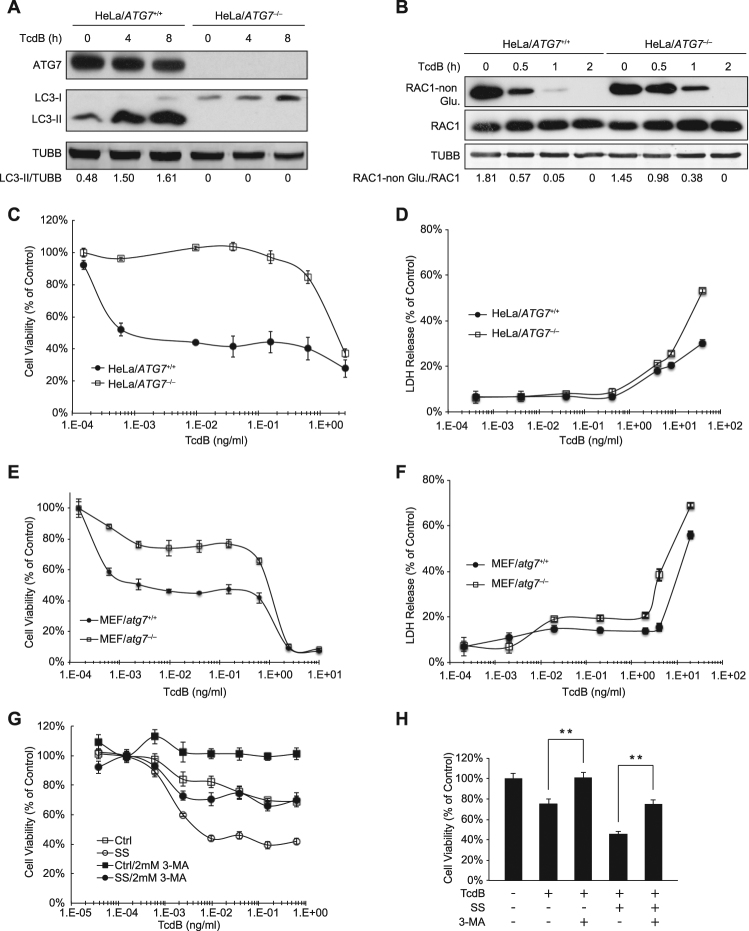
Autophagy induction facilitates TcdB-caused cell proliferation inhibition. (**A**) Effect of ATG7 deficiency on TcdB-induced autophagy in HeLa cells. Wild type and ATG7 knockout (KO, through TALEN technique) HeLa cells were treated by TcdB (1 ng/ml) for 0, 4 or 8 h, before lysed for immunoblotting analysis. (Full-length blots are presented in Supplementary Fig. S12A). (**B**) Effects of ATG7 deficiency on TcdB-induced Rac1 glucosylation in HeLa cells. Wild type and *ATG7* ^−/−^ HeLa cells were exposed to TcdB (5 ng/ml) for indicated period, before lysed for immunoblotting assay to detect the total and non-glucosylated Rac1 protein level. (Full-length blots are presented in Supplementary Fig. S12B). (**C**) Effect of ATG7 deficiency on TcdB-triggered cell viability changes in HeLa cells. Cell viability assay was performed to determine the cytopathic effect of TcdB on wild type and *ATG7* deficient HeLa cells. The values shown represent the mean ± standard deviation (n = 6), as defined by error bars in this and other figures. (**D**) Effect of ATG7 deficiency on TcdB-triggered cell death in HeLa cells. The cells were incubated with TcdB toxin for 48 h before the LDH assay as described in the Experimental Procedures. The values shown represent the mean ± standard deviation (n = 3). (**E**) Effect of ATG7 deficiency on TcdB-triggered cell viability changes in MEFs. The MTT assay was performed as described in the Experimental Procedures. The values shown represent the mean ± standard deviation (n = 6). (**F**) Effect of ATG7 deficiency on TcdB-triggered cytotoxicity in MEFs. The LDH assay was performed to determine the killing effect of TcdB on the wild type and MEF/*atg7* ^−/−^. The cells were incubated with TcdB toxin for 48 h before the LDH assay. The values shown represent the mean ± standard deviation (n = 6). (**G**) Effect of 3-MA on TcdB-triggered cell viability changes in HeLa cells with or without serum starvation treatment. (**H**) The statistic results from (**G**). The data point shown here is under 39.1 pg/ml TcdB exposure for 48 h. **P < 0.01 (n = 6), t-test.

Given that TcdB induced a dramatic autophagy response in host cells, we wanted to know next whether the induced autophagy plays a role in TcdB-mediated cytotoxic or cytopathic effects. To answer this, we generated ATG7 knockout HeLa cells, since ATG7 is essential for the early steps of autophagosome formation. Cells lacking this protein are deficient in conventional autophagy, as demonstrated by the loss of LC3 lipidation^62^. Indeed, HeLa cells with complete loss of ATG7 expression failed to respond to either SS (Supplementary Fig. S3) or TcdB exposure as there was no LC3-I conversion to LC3-II (Fig. 2A). Besides, knockout of ATG7 had little effect in delaying the Rac1 glycosylation that indicates the TcdB endocytosis process (Fig. 2B). From the results of the MTT and LDH assays, it showed that HeLa/*ATG7* ^−/−^ cells were more resistant to TcdB in terms of the inhibition of cell proliferation (Fig. 2C), since the killing activity of TcdB was marginal under this concentration (Fig. 2D), and it was observed that cell growth was highly inhibited after TcdB treatment (Supplementary Fig. S4). The cell viability shown in the MTT analysis started to drop sharply when TcdB dosage exceeded 1ng/ml (Fig. 2C), correlating well with the occurrence of cell death as shown in the lactate dehydrogenase (LDH) assay (Fig. 2D). Consistently, the cell viability and the LDH assays in MEF/*atg7* ^−/−^ showed that the lack of ATG7, an essential gene for autophagosome formation and autophagy induction^62^, led to an increase in cell viability upon TcdB exposure (Fig. 2E). This was due to less inhibition of cell proliferation rather than reduced cell death when the toxin concentration is below 1 ng/ml (Fig. 2F). These data suggested that the ATG7-dependent induction of autophagy plays a critical role in TcdB-mediated cell-growth inhibition.

In addition to the genetic cell models, we also found that the addition of 3-methyladenine (3-MA), an autophagy inhibitor by blocking PI3K function^63^, resulted in increased cell viability in HeLa cells treated with TcdB, while serum starvation could reverse this phenotype caused by 3-MA and made cells more sensitive to TcdB (Fig. 2G,H). Our data indicated that the autophagy pathway is required for TcdB-mediated inhibition of cell proliferation, one of the major cytopathic effects caused by TcdB^55^.

Besides cell growth arrest, our data also showed that TcdB triggered cell death under higher concentrations (Fig. 2D,F), consistent with previous reports demonstrating that high concentrations of TcdB lead to cell death, which has been defined as necrosis^58^. To investigate the role of autophagy in TcdB-triggered cell death, we monitored both cell viability and cell death under the treatment of low or high concentrations of TcdB in autophagy-deficient HeLa and NRK cells. When exposed to low concentrations of TcdB, as expected, both knockout cell lines behaved more resistant to TcdB-caused cell proliferation inhibition. However, under higher concentrations of TcdB, which started to kill host cells, both autophagy-deficient cells became more sensitive to TcdB-caused cell death (Supplementary Fig. S5). These data suggested that autophagy might play a protective role in TcdB-triggered cytotoxicity under higher dosage.

### Glucosyltransferase Activity of TcdB Is Required for Autophagy Induction

As it is well-known that TcdB induces strong autophagy to hinder host cell proliferation, we set out to identify the responsible domain(s) of this toxin. The primary structure of TcdB is divided into the glucosyltransferase (GT) domain, the cysteine protease domain (CPD), and the delivery/receptor-binding domains^64^. Although detailed analyses of the regions outside the enzymatic domain are not clear, it has been established that the GT domain (GTD) consists of the first 543 amino-acids, including a conserved DXD motif that is essential for its glucosyltransferase activity^65^.

**Figure 3:**
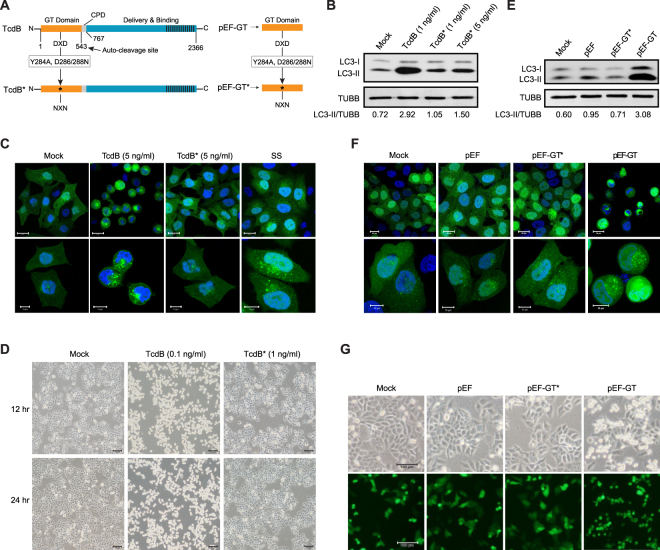
Glucosyltransferase activity of TcdB is required for autophagy induction. (**A**) Multi-domain structure of *C*. *difficile* toxin B (TcdB) and GT domain expression constructs. The DXD motif was mutated to generate a glucosyltransferase-inactive TcdB* (Y284Aand D286/288 N). The glucosyltransferase domain (GT) and its mutant version (GT*) were cloned into pEF expression vector. (**B**) Effect of glucosyltransferase activity on TcdB-induced autophagy. Immunoblotting analysis of LC3 of HeLa cells treated by TcdB (1 ng/ml) or TcdB* (1 or 5 ng/ml) for 12 h, with untreated HeLa cells as control (Mock). (Full-length blots are presented in Supplementary Fig. S13A). (**C**) Effect of glucosyltransferase activity on TcdB-induced autophagy and cell-rounding. Fluorescence microscopy of HeLa cells stably expressing GFP-LC3 treated for 8 h by 5 ng/ml of TcdB or TcdB*, with untreated (Mock) and SS-treated HeLa cells as controls. The lower panels (scale bar = 10 μm) showed higher magnification than the upper panels (scale bar = 20 μm), the statistical summary of the CFP-LC3 positive dots are shown in Supplementary Fig. S6B. (**D**) Cytopathic effect of glucosyltransferase activity of TcdB. The cell-rounding phenotype was observed under light microscope of HeLa cells treated by TcdB (0.1 ng/ml) or TcdB* (1 ng/ml) for 12 or 24 h. Scale bar = 100 μm. (**E**) Effect of GT domain expression on host cells. Immunoblotting analysis of LC3 of HeLa cells transiently transfected by pEF-GT or pEF-GT*, with mock and pEF empty vector as controls. The cell lysates for immunoblotting were harvested 24 h after transfection. (Full-length blots are presented in Supplementary Fig. S13B). (**F**) Fluorescence microscopy of HeLa cells (stably expressing GFP-LC3) transiently transfected by pEF-GT or pEF-GT*, with mock and pEF6-BSD empty vector as controls. The images were taken 24 h after transfection. The lower panels (scale bar = 10 μm) showed higher magnification than the upper panels (scale bar = 20μm). (**G**) Fluorescence microscopy of HeLa cells transiently transfected by mock, pEF vector, pEF-GT or pEF-GT*, all co-transfected with GFP-expressing plasmid pEGFP at a ratio of 10:1 to indicate transfection. The cell-rounding phenotype was observed under both light (top) and fluorescence microscope (bottom) 24 h post transfection. Scale bar = 100 μm.

To clarify whether the GT domain of TcdB and/or its enzymatic activity is involved in the autophagy induction, we expressed and purified a mutant TcdB (TcdB*) protein harboring three point mutations (Y284A and D286/288 N) (Fig. 3A; Supplementary Fig. S6A) that completely abolished the toxin’s glucosyltransferase activity^66^. As shown by both immunoblotting analysis and fluorescence microscopy, TcdB*, unlike its wild type counterpart, failed to induce autophagy response as indicated by GFP-LC3 puncta accumulation and increased endogenous LC3-II level (Fig. 3B,C; Supplementary Fig. S6B). This suggests that the glucosyltransferase activity of TcdB is required for triggering autophagy. As predicted, TcdB* also lost the capability of eliciting the cell-rounding phenotype (Fig. 3C,D), consistent with previous studies^65^.

To further verify whether this enzymatic domain is sufficient to trigger autophagy, plasmids expressing only the GT domain or its corresponding mutant were constructed (Fig. 3A) and introduced into HeLa cells. The autophagosome accumulation and the increase of LC3-II levels were only found in cells expressing the wild type GT domain, but not the mutant GT* (Fig. 3E,F). To confirm that the observed autophagy induction is indeed due to the GT domain expression, we co-transfected the GFP-expressing plasmid with GT- or GT*-expressing plasmid at 1:1 ratio. Twenty-four hours after co-transfection, cells that became rounded were exclusively those expressing GFP and GT, rather than GFP and GT* (Fig. 3G). This result suggested that the glucosyltransferase activity of the GT domain, which is reported to be responsible for the disruption of the cytoskeleton in host cells^65^, is necessary and sufficient to induce autophagy upon TcdB exposure.

### TcdB-induced Autophagy and Cell-rounding Are Not Interdependent

**Figure 4:**
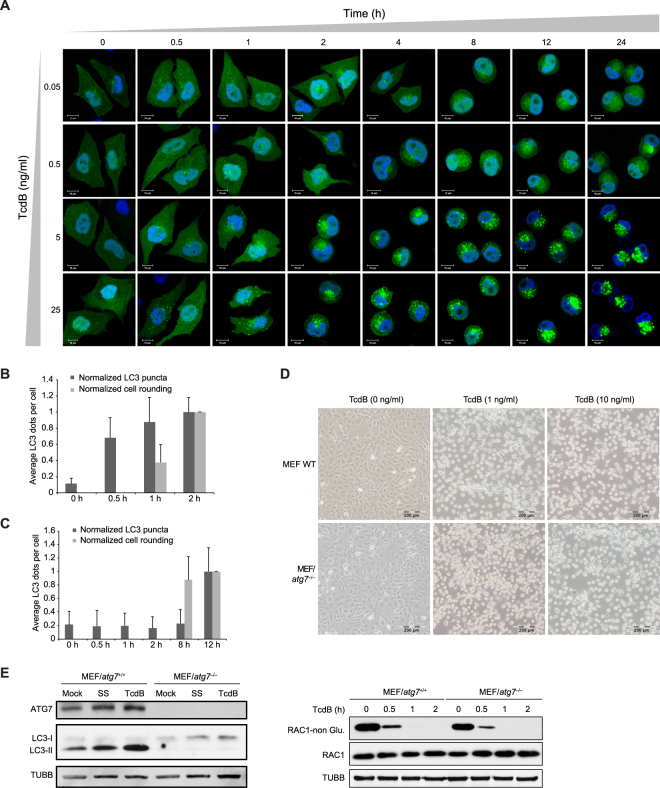
TcdB-triggered autophagy induction and cell-rounding are not interdependent. (**A**) Fluorescence microscopy of HeLa cells stably expressing GFP-LC3 treated by increasing amount of TcdB (from top to bottom), and by increasing time of treatment (from left to right). Scale bar = 10 μm. Representative images of four independent experiments are shown. (**B**) The statistic results of (A) under the exposure of 5 ng/ml TcdB. For each time point, the average number of LC3 puncta per cell and the number of rounding cells were counted from over 50 cells and normalized to the time point of 2 h. (**C**) The statistic results of (**A**) under the exposure of 0.05 ng/ml TcdB. For each time point, the average number of LC3 puncta per cell and the number of rounding cells were counted from over 50 cells and normalized to the time point of 12 h. (**D**) Effect of autophagy deficiency on TcdB-triggered cell-rounding. Light microscopic images of wild type (*atg7* ^+/+^) and *atg7* ^−/−^ MEFs treated by 1 or 10 ng/ml of TcdB for 8 h. Scale bar = 200 μm. (**E**) Effect of ATG7 deficiency on TcdB-induced autophagy (left) and Rac1 glucosylation (right) in MEF cells. Wild type and *atg7* ^−/−^ MEF cells were treated by TcdB (1 ng/ml) or SS (serum starvation) for 8 h, before lysed for immunoblotting analysis (left); Wild type and *atg7* ^−/−^ MEF cells were exposed to TcdB (5 ng/ml) for indicated period, before lysed for immunoblotting assay to detect the total and non-glucosylated Rac1 protein level (right). (Full-length blots are presented in Supplementary Fig. S14).

Since the glucosyltransferase activity is responsible for both TcdB-triggered autophagy and cell-rounding, and it has been reported that extracellular matrix (ECM) detachment can induce autophagy^67^, we investigated whether there is a causal relationship between autophagy induction and cell-rounding. TcdB causes cell rounding through its glucosyltransferase activity by inactivating small GTPases of the Rho family^39, 65, 68^. To address this question, we conducted a detailed fluorescence microscopic assay to investigate the dynamic changes of cell morphology and autophagy induction with exposure to a series of TcdB doses. Interestingly, the autophagy induction, indicated by the increased GFP-LC3 signals, occurred earlier than the appearance of cell-rounding when cells were exposed to relatively high concentrations of toxin (Fig. 4A; Supplementary Fig. S7C,D). This was further confirmed by the statistical analysis of the average number of LC3 puncta in each cell, which showed that the LC3-labelled autophagosomes increased significantly at 0.5 h under the treatment of 5 ng/ml TcdB while most cells did not show the rounded morphology (Fig. 4B). On the contrary, the autophagy induction occurred later than the cell-rounding appearance when cells were incubated in relatively low concentrations of toxin (Fig. 4A; Supplementary Fig. S7A,B). This was proved by the statistical analysis of LC3 puncta in each cell under 0.05 ng/ml TcdB treatment (Fig. 4C). These data ruled out the possibility that cell-rounding is a prerequisite for autophagy induction. Furthermore, TcdB was able to trigger cell-rounding morphology in autophagy-deficient MEF/*atg7* ^−/−^ cells as efficiently as in wild type cells (Fig. 4D), as well as inducing similar glucosylation rates of the Rac1 substrate (Fig. 4E). Thus, we concluded that TcdB-triggered cell-rounding and autophagy are not interdependent.

### Both the mTOR Pathway and PI3K Complex Are Involved in the TcdB-induced Autophagy Process

**Figure 5:**
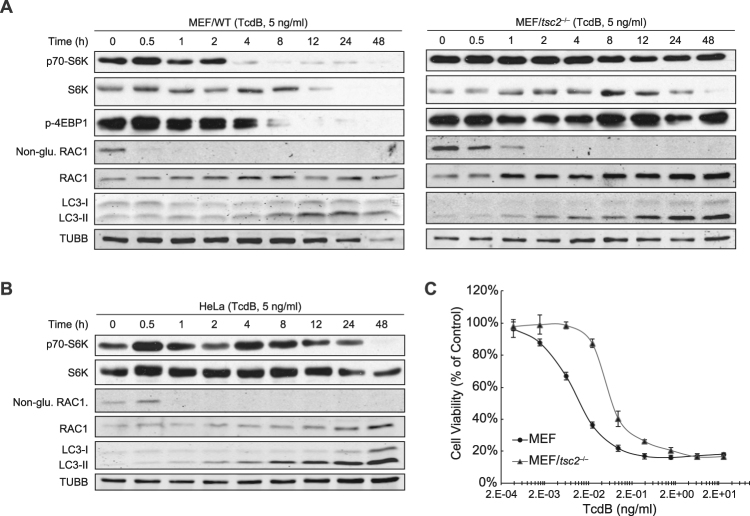
mTOR is involved in but not required for TcdB-induced autophagy. (**A**) Immunoblotting analysis for the effect of TcdB on wild type (left) and *tsc2* ^−/−^(right) MEFs. Cells were treated with TcdB (5 ng/ml) for indicated time, before lysed for the western blotting. The phosphorylation of mTOR substrates (S6K and 4EBP1), the glucosylation of TcdB substrate (Rac1), and the turnover of LC3 were detected by their corresponding antibodies. The detailed methods were described in Experimental Procedure. (Full-length blots are presented in Supplementary Fig. S15). (**B**) Immunoblotting analysis for the effect of TcdB (5 ng/ml) on HeLa cells. The experiment procedure was the same as above (**A**). The quantification of this figure is shown in Fig. S5C. (Full-length blots are presented in Supplementary Fig. S16). (**C**) Cytotoxicity of TcdB on wide type and MEF/*tsc2* ^−/−^. Cell viability assay was performed to determine the cytotoxicity of TcdB on wild type and MEF/*tsc2* ^−/−^. The MTT assay was performed as described in the Experimental Procedures. The values shown represent the mean ± standard deviation (n = 6), as defined by error bars.

Based on the role of autophagy in the TcdB-triggered cytopathic process, the next step was to determine the key factors or signaling pathways of the autophagy process responsible for this functional phenotype. The mammalian target of rapamycin, mTOR, a serine/threonine kinase, is a potent suppressor of autophagy, mediating a variety of stimuli to regulate the autophagic process^4, 5, 60^. As a negative upstream regulator of mTOR, TSC2 is responsible for sensing the cellular amino acid contents indirectly, and inhibiting mTOR signaling^6, 7^. To demonstrate whether mTOR participates in TcdB-induced autophagy, wild type and *tsc2* ^−/−^ MEFs were used to assess the effects of TcdB exposure. Upon treatment of 5 ng/ml TcdB in MEFs, most of Rac1, one of the glucosyltransferase substrates, was converted to glycosylated form within 30 min. The suppression of mTOR occurred after 4–8 h, as indicated by the reduced phosphorylation of S6K and 4EBP1, two surrogate makers for mTOR kinase activity^69^, and lipidated LC3-II also appeared (Fig. 5A left). In MEF/*tsc2* ^−/−^ (Fig. 5A right), TcdB no longer caused the reduction of the phosphorylation of S6K and 4EBP1, suggesting that TcdB-triggered mTOR inactivation observed in the wild type MEFs was conducted through TSC2. However, the conversion of LC3-I to LC3-II still occurred in MEF/*tsc2* ^−/−^. TcdB was still able to glycosylate Rac1 in MEF/*tsc2* ^−/−^, although this process was delayed by 0.5–1 h.

Similar results were obtained in HeLa cells, with the TcdB-triggered inhibition of mTOR activity occurring after 8 h as indicated by the decrease of p70-S6K (Fig. 5B), although there was an increase at 4 h. This phenomenon occurred in HeLa cells that were exposed to all three concentrations of TcdB, 5 ng/ml (Fig. 5B), 0.05 ng/ml (Supplementary Fig. S8A) and 0.5 ng/ml (Supplementary Fig. S8B), with a transient fluctuation (Supplementary Fig. S8C). Consistent with MEF/*atg7* ^−/−^, MEF/*tsc2* ^−/−^ also had a higher resistance to TcdB-triggered cell growth inhibition than wild type (Fig. 5C). These data demonstrated that the TSC2-mTOR pathway is involved in TcdB-induced autophagy and cytopathic effects, although it’s not indispensable for the TcdB-induced autophagy process.

**Figure 6:**
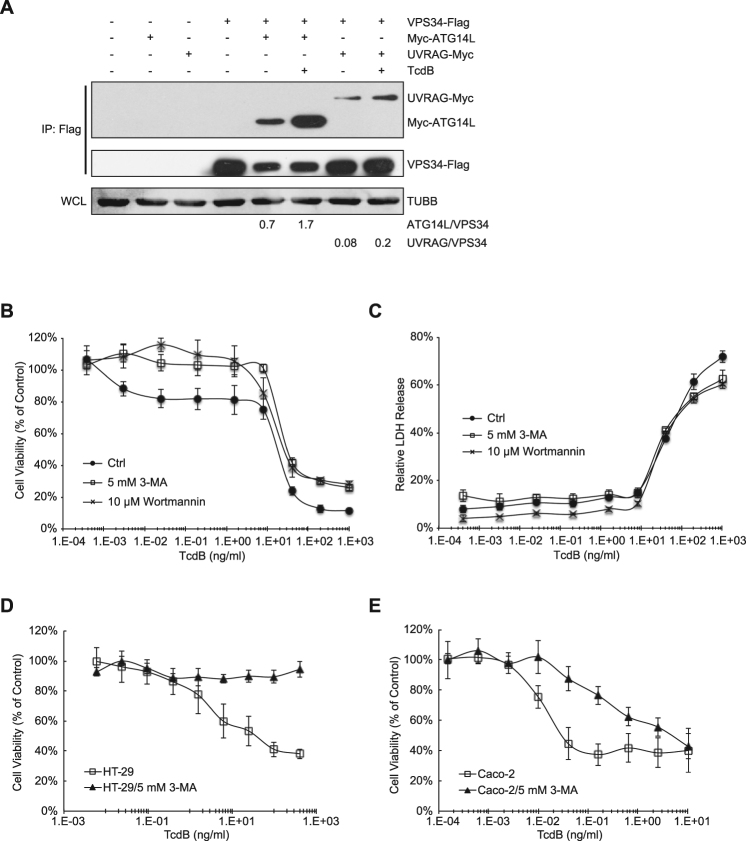
PI3K complex participates in the induction of TcdB-triggered autophagy. (**A**) The protein-protein interaction between VPS34 and ATG14L or UVRAG with or without TcdB treatment. HeLa cells were co-transfected with the plasmids encoding VPS34-Flag and myc-ATG14L/myc-UVRAG. 24 h after transfection, cells were treated with or without TcdB (1 ng/ml) for 3 h, before lysed by NP40 lysis buffer. Cell lysates were immunoprecipitated with anti-Flag mAb and immunoblotted using anti-myc mAb or anti-Flag mAb. The amount of β-tubulin in the whole cell lysate (WCL) was assayed as the loading control. The intensity of the blotting signal was quantified with ImageJ (http://rsbweb.nih.gov/ij/), and the relative intensity was labeled below the images. (Full-length blots are presented in Supplementary Fig. S17). (**B**) Effect of 3-MA and wortmannin on TcdB-triggered cell viability changes in HeLa cells. The MTT assay was performed in HeLa cells treated by a series concentration of TcdB in the presence or absence of PI3K inhibitor, 3-MA (5 mM) or wortmannin (10 μM). The values shown represent the mean ± standard deviation (n = 6), as defined by error bars. (**C**) Effect of 3-MA and wortmannin on TcdB-triggered cell death in HeLa cells. The LDH cytotoxicity assay was performed as described in the Experimental Procedures. The values shown represent the mean ± standard deviation (n = 3). (**D** and **E**) Effects of 3-MA on TcdB-triggered cytotoxicity on HT-29 and Caco-2 cells. The MTT assay was performed as described in Experimental Procedures to determine cell viability of HT-29 cells (**D**) and Caco-2 cells (**E**) under TcdB treatment. The values shown represent the mean ± standard deviation (n = 6), as defined by error bars. One of at least three experiments is shown.

Besides the role of mTOR in the regulation of autophagy, we also wanted to know if the toxin triggers this process through the class III PI3K complex, a key complex for autophagy initiation^70^. The typical PI3K complex contains VPS34, VPS15, BECN1, and ATG14L or UVRAG^8, 71^. To verify whether the PI3K complex is involved in TcdB-mediated autophagy induction, we studied the effects of TcdB on the protein-protein interaction amongst components of the PI3K complex. After co-introduction into HeLa cells, Flag-tagged VPS34 specifically precipitated with Myc-tagged ATG14L and UVRAG, confirming that VPS34 was able to bind to these two proteins^71, 72^. Interestingly, TcdB treatment significantly increased the amount of Myc-tagged ATG14L and UVRAG precipitated by Flag-tagged VPS34 (2–3 folds’ increase) in the co-immunoprecipitation assay (Fig. 6A). These results showed that TcdB enhances the PI3K complex formation, which includes at least VPS34, and ATG14L or UVRAG. In agreement with the above results, the addition of VPS34 inhibitors, 3-methyladenine (3-MA) and wortmannin^73^, inhibited TcdB-induced cell proliferation inhibition (Fig. 6B,C). Similar results were obtained from HT-29 and Caco-2 cells. 3-MA treatment provided cell resistance to TcdB-triggered cell proliferation inhibition (Fig. 6D,E), further supporting a universal role of the PI3K complex in TcdB-induced autophagy and consequently cell growth inhibition. These data indicated that both the mTOR pathway and the PI3K complex play important roles in TcdB-induced autophagy and consequently cytopathic effects through cell growth inhibition.

## Discussion

**Figure 7:**
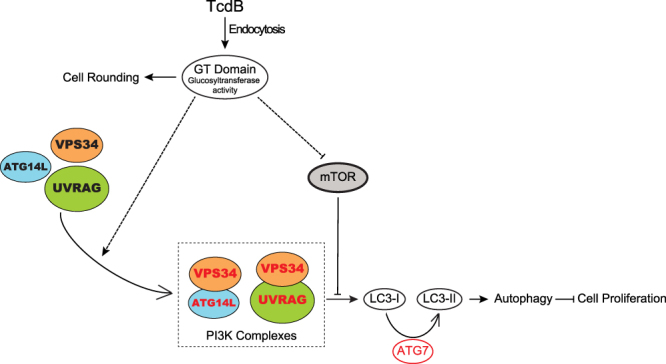
Model of TcdB-mediated autophagy induction and cell-growth inhibition. After TcdB enters host cells, its GT domain is released into the cytoplasm, and subsequently glucosylates and inactivates the Rho family small GTPases and/or other substrates, resulting in the disruption of cell cytoskeleton and cell-rounding. The glucosyltransferase activity of TcdB, through an uncharacterized mechanism, directly or indirectly, enhances the formation of PI3K complex that includes VPS34 and ATG14L or UVRAG, and inhibits the activity of mTOR pathway, to induce high level of autophagy in host cells. The excessive level of this self-eating process could facilitate toxin-triggered cell proliferation inhibition.

Autophagy is an important self-eating process involved in microbial pathogenicity. Here we report for the first time that host autophagy can be triggered by *C*. *difficile* cytotoxin B through its glucosyltransferase activity. The autophagy response affects TcdB’s cytopathic effects by facilitating TcdB-caused cell proliferation inhibition. Our mechanistic studies indicate that the glucosyltransferase activity-related cell-rounding process has no causal relationship with TcdB-triggered autophagy induction, while the mTOR pathway and PI3K complex are involved in this induction process. Based on these results, we provide a working model for host autophagy in the pathogenesis of a glucosyltransferase bacterial toxin (Fig. 7).

The glucosyltransferase activity of TcdB was found necessary and sufficient for TcdB-induced host autophagy (Fig. 2). Thus we speculated that the substrates of TcdB might be responsible for the autophagy induction process. The glucosyltransferation of Rho family proteins by TcdB leads to their irreversible inactivation, which causes cytoskeletal disruption and cell-rounding morphology^39^. However, cell-rounding, which has been reported to be a trigger of autophagy in other cases^67^, is proven not to be the reason of autophagy induction in this case because autophagosome accumulation could occur before cell-rounding under high dosage of TcdB exposure (Fig. 4A,B; Supplementary Fig. S7C,D). It’s still an open question whether the TcdB glucosyltransferased-Rho family proteins directly trigger host autophagy.

It is also possible that other substrates or the overall glucosyltransferased-modification pattern of all substrates of the GTD are recognized by certain autophagy receptors. One of the autophagy receptor candidates is galectin 8. It recognizes and binds to host glycans when exposed to damaged bacteria vacuoles, resulting in the recruitment of NDP52, and the subsequent induction of autophagy through LC3^74^. To investigate the linkage mechanism between TcdB and these proteins, we have tested the interaction and function of some autophagy adaptors (such as NDP52 and SQSTM1/p62) for TcdB-induced autophagy, but none of them gave us a positive result. The modification of these small GTPases by the GTD of TcdB using UDP-glucoses as co-substrates^39^, is uncommon in host cells^75^. Therefore it is still an open question if this unique modification could be recognized by the host as a sign of danger, and in turn inducing autophagy as a self-defense mechanism. How the glucosylation activity of TcdB contributes to these downstream processes remains to be determined.

Once host autophagy is triggered through GT activity, TcdB could maintain the autophagy activity of host cells at a relatively high level (Fig. 1A), a quite different pattern from the case of starvation stimulation, where the autophagy activity fluctuates like a sine wave due to the feedback signaling pathway^1, 76, 77^. mTOR is known to serve as a sensor to monitor the intracellular levels of nutrients^4, 5^, excessive levels of which can activate mTOR to suppress autophagy, providing a negative feedback loop to prevent cell death from excessive autophagy induction^78^. Our results show that the activity of mTOR was gradually decreased by TcdB treatment, with an increase at 0.5 h and 4 h (Fig. 5B; Supplementary Fig. S8). It could be speculated that the transient up-regulation of mTOR activity during the fluctuation cycle may be a result of the feedback mechanism, and it is eventually overpowered by the inhibition mediated by TcdB. This suggests that the feedback signaling pathway was somehow turned off by TcdB at a later stage.

In many infection cases, the host autophagy interacts with the pathogen endocytosis process. For instance, autophagy facilitates the cytoplasmic delivery of ANTXR2-associated LF during endocytosis^79^. In the case of TcdB exposure, however, the induction of host autophagy seems to be unrelated to endocytosis of the toxin, based on the following evidence: (1) the mutant TcdB without its glucosyltransferase activity^65, 66^ failed to elicit a autophagy response (Fig. 3A–C; Supplementary Fig. S6B); (2) the efficiency of TcdB in glucosylating the substrate Rac1, and causing the cell-rounding phenotype were similar in both wild type and autophagy-null mutants (*atg7* ^−/−^) MEFs (Fig. 4D,E); (3) the intracellular expression of the GT domain of TcdB through plasmid transfection was sufficient to up-regulate autophagy (Fig. 3E,F).

As in the innate defense mechanism, autophagy also functions as a cellular defense mechanism against infection, such as in the case of *Vibrio cholera* cytolysin (VCC) intoxication or anthrax infection, demonstrating a cellular defense role of autophagy against secreted bacterial toxins^21, 80–82^. In addition, the induction of autophagy can overcome the blockade of mycobacterial phagosome maturation, and inhibit the survival of intracellular *Mycobacterium tuberculosis* ^83^. In order to evade autophagy-mediated defense mechanisms, many microbial pathogens or their virulence factors have evolved a myriad of strategies. For instance, *Listeria monocytogenes* uses a variety of mechanisms to evade destruction by the host autophagy system in order to colonize the cytosol of macrophages^84^. Also, cAMP-elevating toxins suppress immune responses, and modulate host cell physiology by inhibiting host autophagy^85^. The case we have presented here shows that autophagy induction facilitates TcdB-induced cell proliferation inhibition under the exposure of low-dosage TcdB, while it blocks cell death triggered by a high-dosage of TcdB (Fig. 2; Supplementary Fig. S5). There are two explanations to interpret this phenotype: 1) Autophagy plays as an accomplice of the toxin to facilitate its inhibition of cell proliferation, which might cause some pathogenic effects such as inflammation. This process could be beneficial for the bacteria in terms of the convenience in spreading and acquiring host nutrients. 2) Autophagy plays a protective role by inhibiting cell death, so as to fight against TcdB-induced pathogenic effects upon high concentrations of TcdB exposure. The physiological concentrations of *C*. *difficile* toxin B around local tissue cells, however, still remain a controversial question^58, 86–88^. We believe that autophagy participates in the pathogenesis of *C*.*difficile* infection as either an “accomplice” or a “protector” to influence TcdB-triggered pathogenic effects.

In summary, we have demonstrated for the first time that a glucosylation enzyme originated from a pathogenic bacterium triggers a strong host autophagy response and related pathogenesis processes. These findings support a new working paradigm for the relationship between autophagy and microbial pathogenesis.

## Methods

### Cell lines and medium

HeLa, HT-29 and MEF cells were cultured in Dulbecco’s Modified Eagle’s Medium (Gibco #12800-017) containing 10% fetal bovine serum (Hyclone #SN30087.02). Caco-2 cells were cultured in Minimum Essential Medium (Gibco #41500-034) containing 10% fetal bovine serum. MEF/*tsc2* ^−/−^^89^ and MEF/*atg7* ^−/−^^62^ with their corresponding control cells were kindly provided by Drs. Hongbing Zhang (Peking Union Medical College) and Masaaki Komatsu (Tokyo Metropolitan Institute of Medical Science), respectively. GFP-LC3 expressing HeLa cell line was obtained through transfection and G418 (800–1000 μg/ml) (Sigma #A1720) selection.

### Antibodies and reagents

We used the following primary antibodies: monoclonal antibodies (mAbs) against LC3 (M186-3) and ATG7 (PM039) from MBL, phospho-p70S6 kinase (#9206), p70S6 kinase (Thr389, #9202), phospho-4E-BP1 (Thr37/46, #9459), Myc-Tag (71D10) rabbit mAb (#2278 s) from Cell Signaling Technology. Rac1-102 mAb (#610650) from BD Transduction Laboratories was used to specifically detect the non-glucosylated Rac1 (Non-glu. Rac1), and Rac1 mAb 23A8 (#05-389) from Millipore was used to detect the total Rac1. Monoclonal anti-Flag-HRP (#A8592) was ordered from Sigma. The secondary antibodies HRP-conjugated goat anti-mouse IgG (H+L) (#115-035-003) and HRP-conjugated goat anti-rabbit IgG (H+L) (#111-035-003) were purchased from Jackson Immuno Research. The following chemicals were used: chloroquine (CQ), 3-Methyladenine (3-MA, M9281) and wortmannin (W1628) from Sigma, and 3-^90^-2,5-diphenyltetrazolium bromide (MTT) from Amresco.

### Plasmids

The GFP-LC3 construct was kindly provided by Dr. Li Yu (Tsinghua University). pCMV-myc-ATG7 (24921) and pcDNA4-VPS34-Flag (24398) were purchased from Addgene. pEF-ATG14L, pEF-UVRAG were constructed onto pEF6-BSD-myc/his-B vector. pHis1522-TcdB plasmid was kindly provided by Dr. Hanping Feng (University of Maryland). pHis1522-TcdB-mutant (Y284A &D286N/D288N) was cloned by site mutagenesis. pEF6-BSD-B was used to construct plasmids expressing either glucosyltransferase domain of TcdB or its mutant form (Y284A &D286/288 N). pCMV5-3xFlag-ATG7 was constructed into pCMV5 vector. Primers and other information are listed in supplementary materials.

### Fluorescence microscopy

HeLa and HT-29 cells were grown onto glass coverslips in 6-well plates. After toxin exposure, cells were washed by PBS twice, fixed by 4% (wt./vol.) paraformaldehyde in PBS for 10 min, and permeabilized in 0.2% Triton X-100 in PBS for 10 min. The coverslips were mounted onto slides using mounting medium containing DAPI solution (Vector Laboratories, Vectashield), and the cells were examined by either LSM 510 laser-scanning confocal microscope (Zeiss) or TCS SP2 spectral confocal system (Leica).

### Cell viability assay

HeLa, HT-29 or Caco-2 cells were seeded in 96-well plates 1 day before the addition of serially diluted TcdB toxin. MTT staining and detection were performed as described^91^. The starting concentration of cells used was 5 × 10^4^ /ml for HeLa, 1 × 10^5^ /ml for both HT-29 and Caco-2 cells. The cells were incubated with TcdB toxin for 48 h at 37 °C before the MTT assay. The spectrophotometer readings at 570 nm were determined using a Multi-Detection Reader (TECAN, Infinite M200). Cell viability was normalized to wells with mock treatment. Each data point and related error bar shown in figures for MTT assays represent the average results from six wells.

### LDH cytotoxicity assay

HeLa, MEF cells were seeded in 96-well plates 8 hours before the addition of serially diluted TcdB toxin. LDH staining and detection were performed as described in product instructions (G1780, Promega). The starting concentration of cells used was 5,000 cells per well and the cells were incubated with TcdB toxin as indicated time at 37 °C before the LDH assay. The spectrophotometer readings at 490 nm were determined using a Multi-Detection Reader (TECAN, Infinite M200). The death signal represented by the amount of LDH release was normalized to wells with maximum LDH activity of total lysed cells. Each data point and related error bars shown in figures for LDH assays represent the average results from three repeats.

### Immunoblotting analysis and co-immunoprecipitation (Co-IP)

For immunoblotting, cells were treated by TcdB, serum starvation treatment (SS), or H_2_O_2_ with or without chloroquine (CQ) for different time periods, and then lysed for immunoblotting analysis according to standard protocol. For Co-IP assay, plasmids were transfected into HeLa cells prior to different treatment, and the cells were subjected for immunoprecipitation analysis according to manufacturer’s protocol of Sigma anti-Flag M2 affinity gel (#A2220, Sigma). Quantitative analysis was performed using ImageJ (http://rsbweb.nih.gov/ij/).

### Construction of stable knockout cell lines using TALENs technique

The design and assembly of the two pairs of TALENs constructs used for *ATG7* gene-knockout were based on our own ULtiMATE protocol^92^. More specifically, the two targeting sequences for *ATG7* loci are 5′-CCTGGACTCTCTAAA-3′ for TALEN^L^ (ATG7) and 5′-CCAAGGCACTACTAA-3′ for TALEN^R^ (ATG7), with a spacer sequence (5′-ctgcagtttgcccctt-3′). The identification and verification of gene knockout events were based on both sequencing analysis of genome PCR fragments of targeting loci and immunoblotting analysis using antibodies specifically against ATG7.

## Electronic supplementary material

Supplementary information
