# Supplementary material for: Glucosyltransferase Activity of Clostridium difficile Toxin B Triggers Autophagy-mediated Cell Growth Arrest
Source: Sci Rep. 2017 Sep 5;7:10532. doi: 10.1038/s41598-017-11336-4 (PMC5585374; doi:10.1038/s41598-017-11336-4)
Supplement: Supplementary file 1 — Supplementary information [file 41598_2017_11336_MOESM1_ESM.pdf]

Glucosyltransferase Activity of *Clostridium difficile* Toxin B Triggers Autophagy-mediated Cell Growth Arrest

Ruina He, Jingyu Peng, Pengfei Yuan, Junjiao Yang, Xiaoji Wu, Yinan Wang and Wensheng Wei

Biomedical Institute for Pioneering Investigation via Convergence (BIOPIC), Beijing Advanced Innovation Center for Genomics, Peking-Tsinghua Center for Life Sciences, State Key Laboratory of Protein and Plant Gene Research, School of Life Sciences, Peking University, Beijing 100871, China

Correspondence should be addressed to W.W. (wswei@pku.edu.cn).

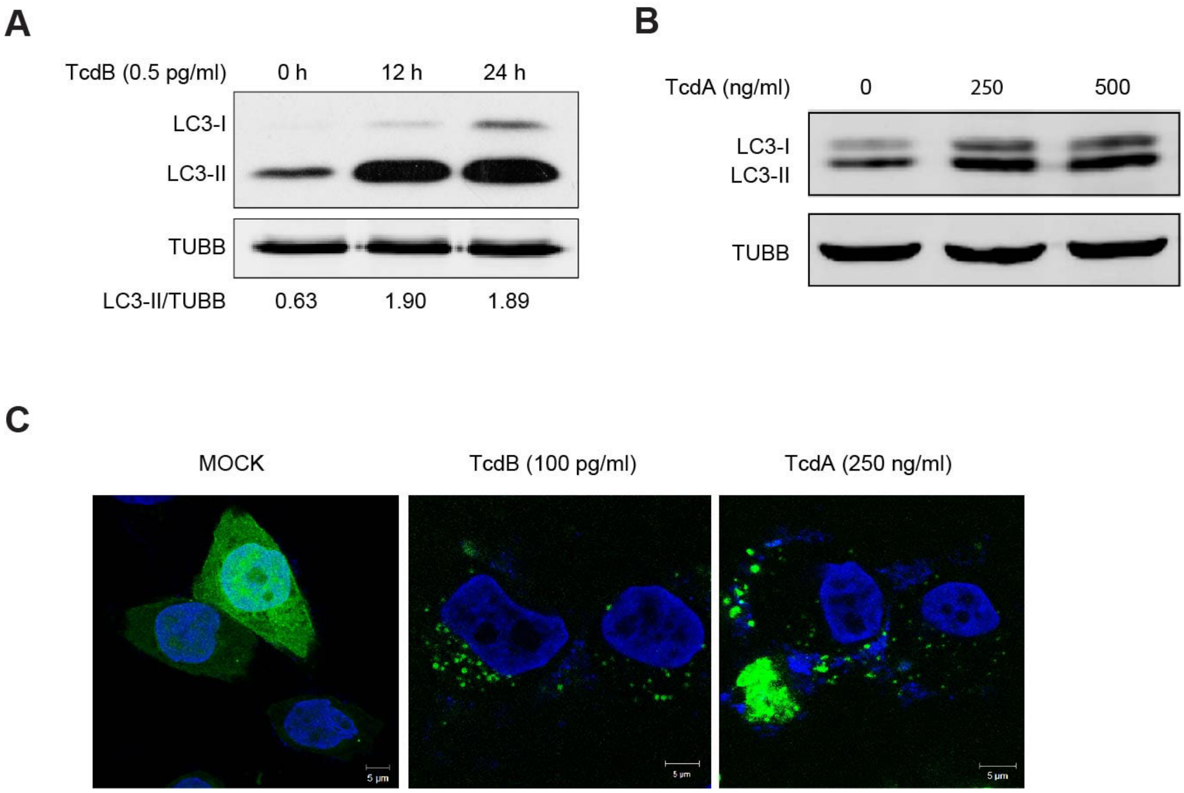

Figure S1. Autophagy Induction by Extremely Low Concentration of TcdB and TcdA

(A) HeLa cells were treated by 0.5 pg/ml TcdB for 12 or 24 h, and lysed for immunoblotting analysis to detect the turnover of LC3-I to LC3-II. All bands of immunoblotting were calculated by Image J. (B) HeLa cells were treated by TcdA as indicated concentration for 12 h, then lysed for immunoblotting analysis. (C) Fluorescence microscopy of HeLa cells stably expressing GFP-LC3 treated by indicated TcdA or TcdB for 12 h.

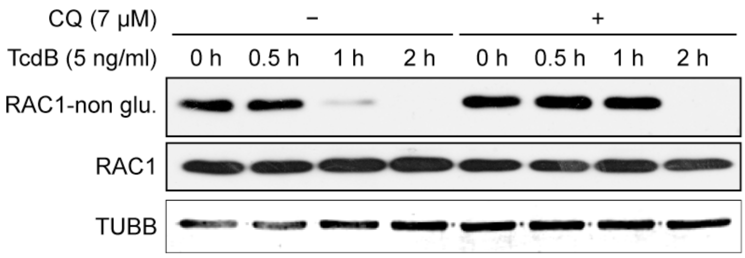

Figure S2. Effects of CQ Treatment on the Endocytosis of TcdB

HeLa cells were treated by 5 ng/ml TcdB for different time periods with or without CQ (7 μM), and lysed for immunoblotting analysis for the detection of Rac1 glucosylation rate.

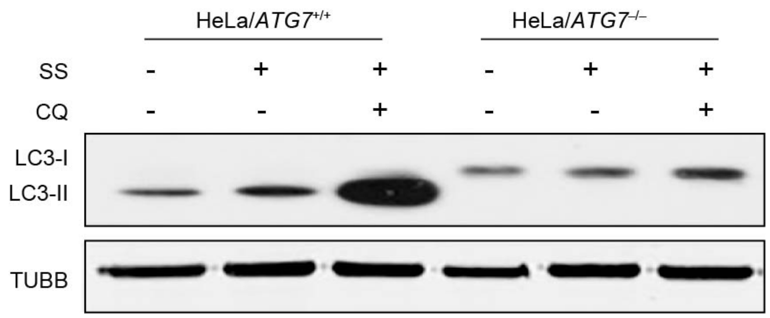

Figure S3. Autophagy Induction in ATG7 Knockout HeLa cells by Serum Starvation.

HeLa cells were treated by serum starvation (SS) for 12 h with or without CQ (7μM), and lysed for immunoblotting analysis to monitor the LC3 turnover.

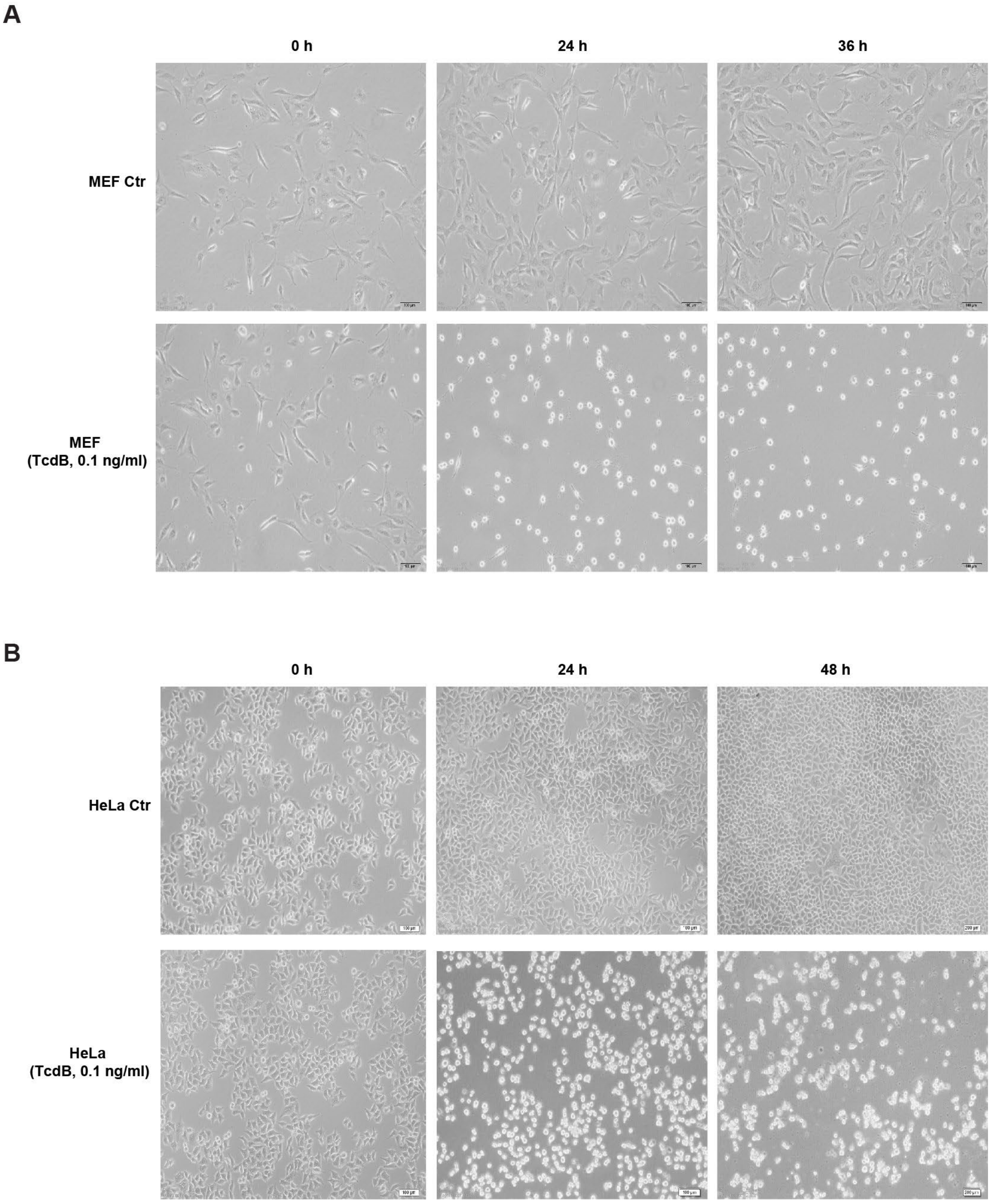

Figure S4. The Host Cell Growth was Inhibited by TcdB.

(A and B) MEF cells (A) or HeLa cells (B) were seeded on 6 well plates. After 12 h, cells were treated with or without TcdB (0.1 ng/ml) for indicated time points. The images were taken by microscope randomly (scale bar = 100 µm).

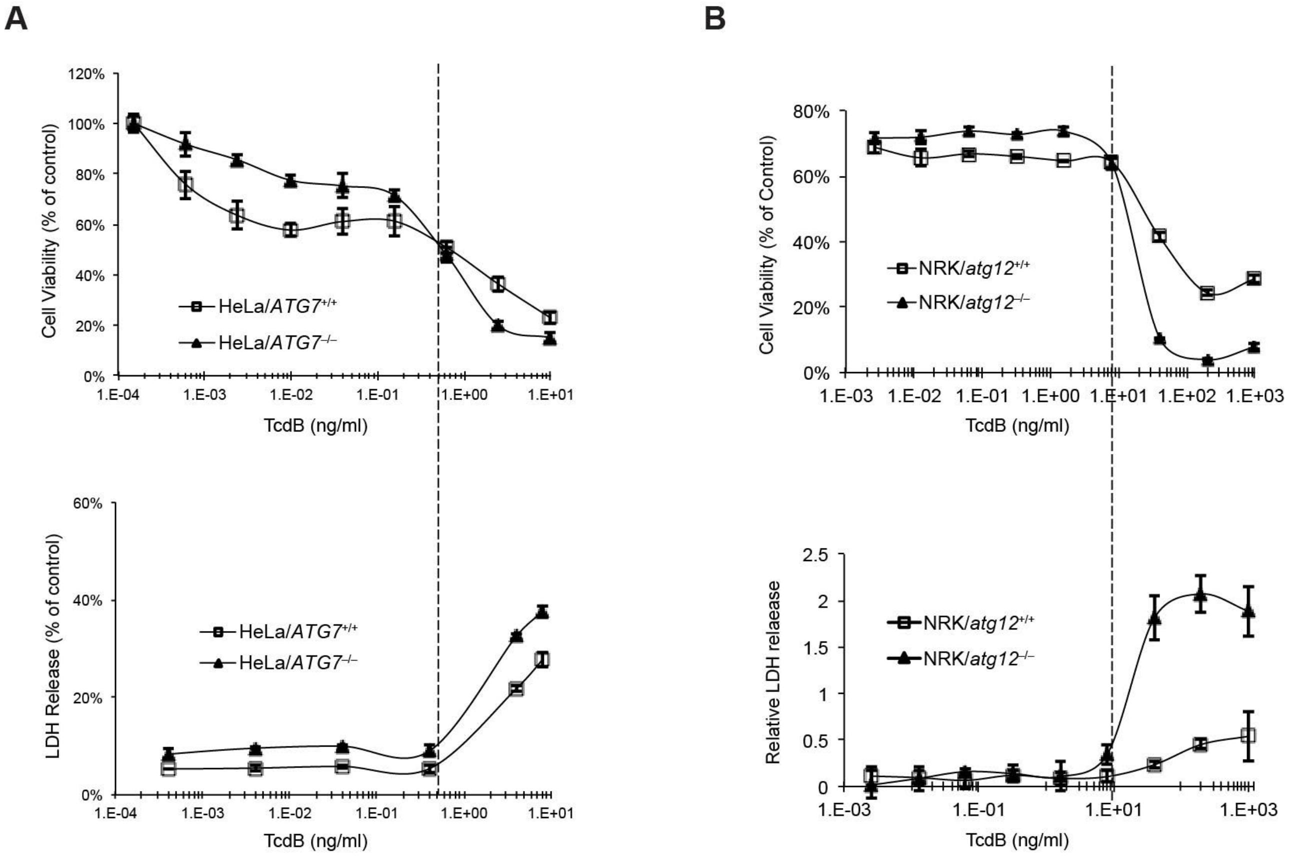

Figure S5. Autophagy Induction Inhibits High Concentration of TcdB-caused Cell Death

(A) Effect of ATG7 deficiency on TcdB-triggered cell viability changes in HeLa cells. Both MTT assay (upper) and LDH assay (bottom) were performed to determine the cytotoxic effect of TcdB on wild type and *ATG7*$^{-/-}$ HeLa cells after cells incubating with TcdB for 48 h. The values shown in the MTT assay represent the mean ± standard deviation (n=6). The LDH cytotoxicity assay was performed as described in the Experimental Procedures. The values shown in LDH assay represent the mean ± standard deviation (n = 3). (B) Effect of ATG12 deficiency on TcdB-triggered cell viability changes in NRK cells. Both MTT assay (upper) and LDH assay (bottom) were performed to determine the cytotoxic effect of TcdB on wild type and *atg12*$^{-/-}$ NRK cells after cells incubating with TcdB for 24 h.

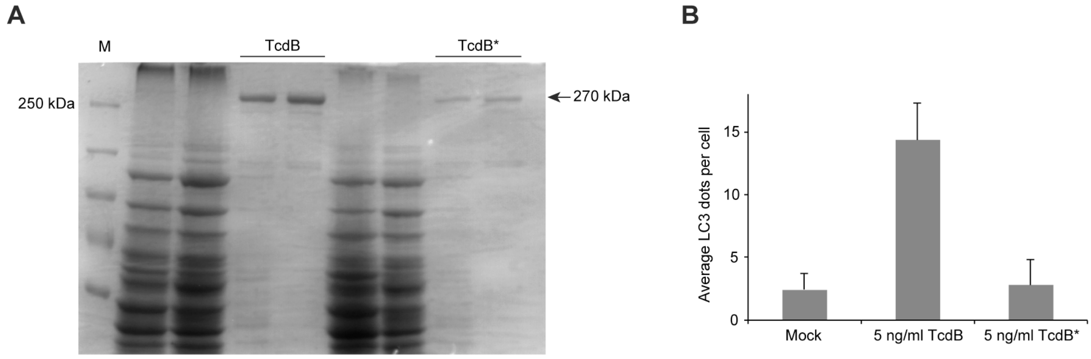

Figure S6. Purification of TcdB and Mutant TcdB* (Y284A& D286/288N) and the Statistical Summary of the GFP-LC3 Dots in TcdB/TcdB*-treated Cells.

(A) Production and purification of wide type TcdB and mutant TcdB (TcdB*). pHis-TcdB and pHis-TcdB* plasmid were transformed into *B. megaterium* protoplasts according to published protocol [1]. The purification was done by His-affinity column from total lysates of *B. megaterium.* (B) The average LC3 dots per cell (images in Fig. 3C) under the treatment of wide type TcdB and mutant TcdB (TcdB*). For each group, the average number of LC3 puncta per cell was counted from over 50 cells.

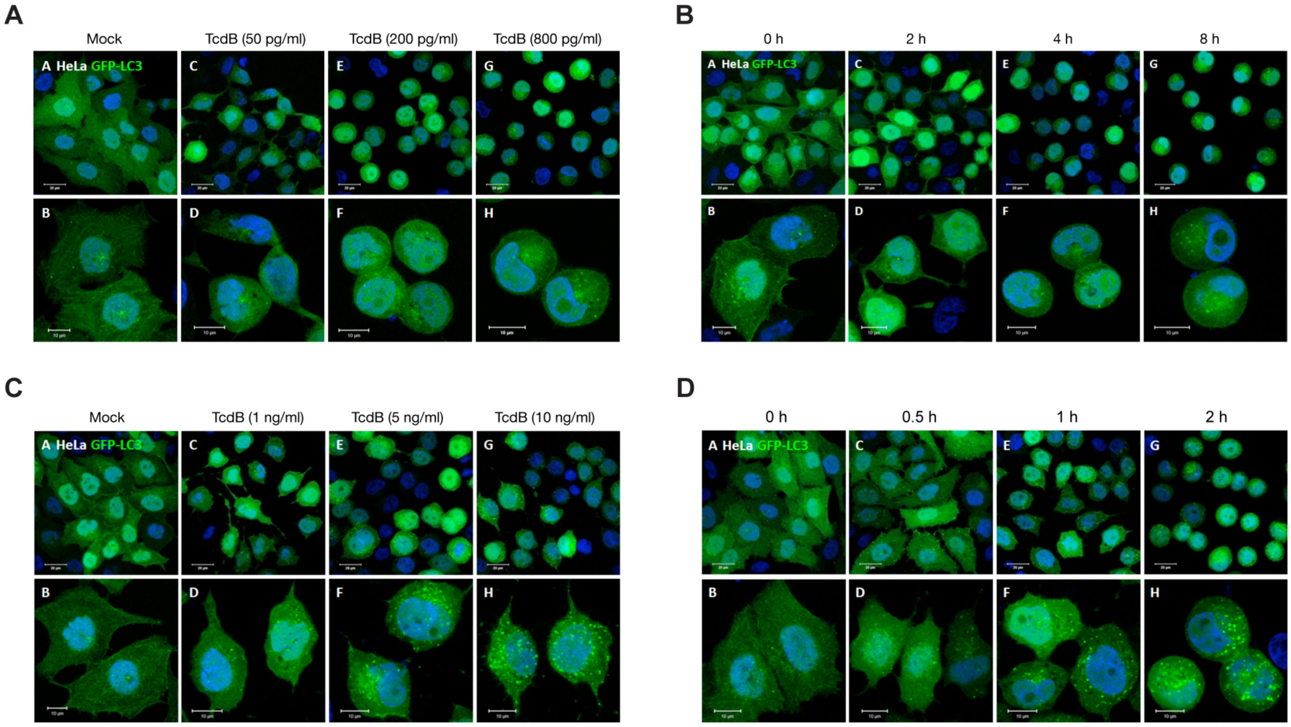

Figure S7. TcdB-triggered Autophagy Induction and Cell Rounding Are Not Interdependent

(A) Fluorescence microscopy of HeLa cells stably expressing GFP-LC3 treated by increasing amount of TcdB (low dosage) for 4 h. The lower panels (scale bar = 10 μm) show higher magnification than the upper panels (scale bar = 20 μm) for this and other panels of this figure. (B) Fluorescence microscopy of HeLa cells stably expressing GFP-LC3 treated by 200 pg/ml of TcdB (low dosage) for increasing amount of time. (C) Fluorescence microscopy of HeLa cells stably expressing GFP-LC3 treated by increasing amount of TcdB (high dosage) for 1.5 h. (D) Fluorescence microscopy of HeLa cells stably expressing GFP-LC3 treated by 5 ng/ml of TcdB (high dosage) for increasing amount of time.

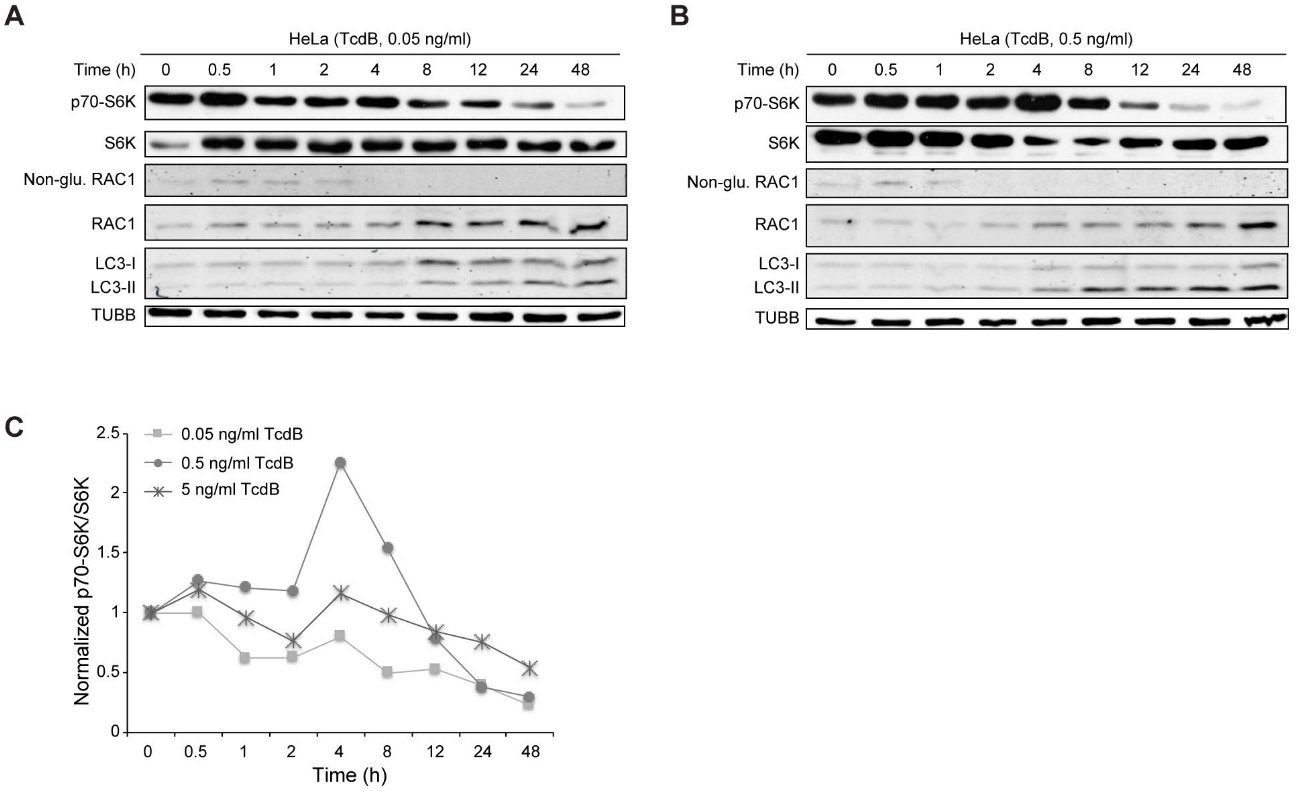

Figure S8. mTOR is Involved in But Not Required for TcdB-induced Autophagy.

(A and B) Immunoblotting analysis for the effect of TcdB on HeLa cells. Cells were treated with 0.05 ng/ml (A) or 0.5 ng/ml (B) of TcdB for indicated time. The amounts of variable proteins were analyzed using indicated antibodies following the protocol as described in Experimental Procedures. (C) The quantification analysis of p70-S6K and S6K in HeLa cells under the treatment of three indicated concentrations of TcdB (responding to Fig 5B, S6A-B). The brightness of p70-S6K and S6K bands was quantified by Image J. The ratio of p70-S6K/S6K was calculated and normalized to time point 0 h.

Figure S9 to Figure S17 displayed full-length blots of cropped WB results used in the main figures.

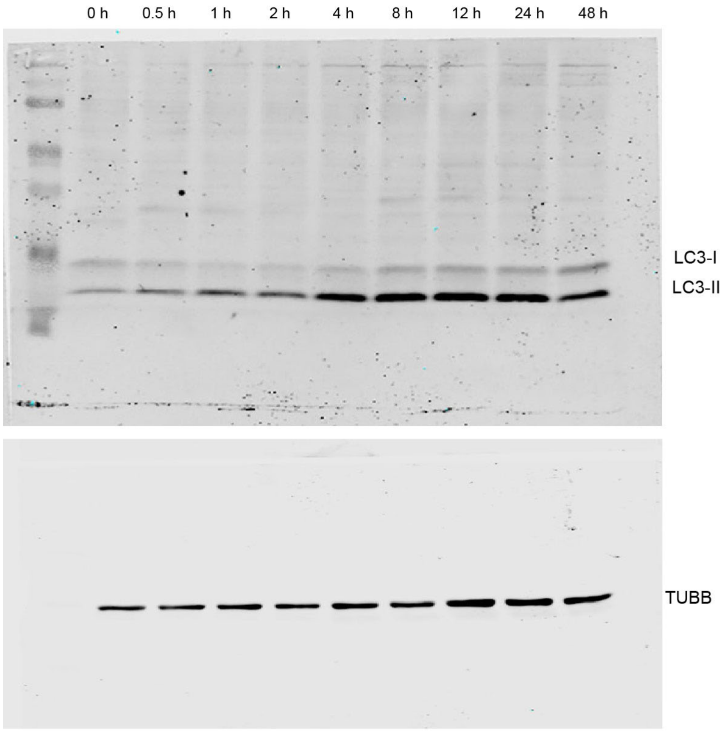

Figure S9. Autophagosome accumulation with TcdB treatment in HeLa cells. (Full length blot of Figure 1C)

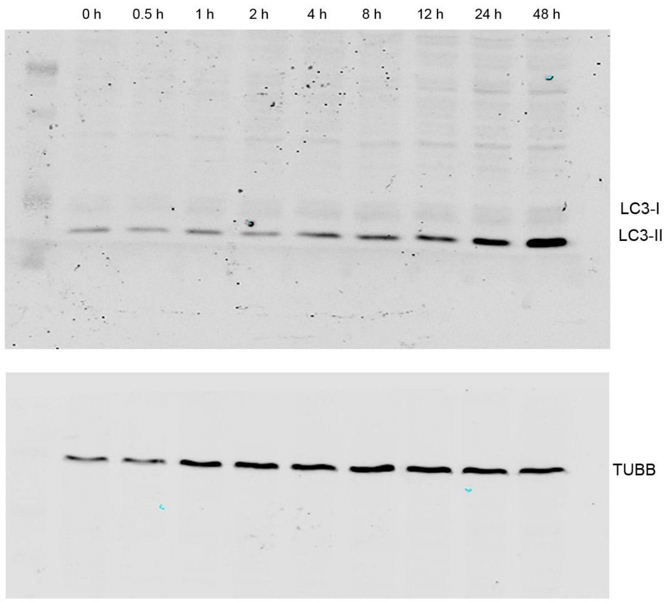

Figure S10. Autophagosome accumulation with TcdB treatment in HT-29 cells. (Full length blot of Figure 1H)

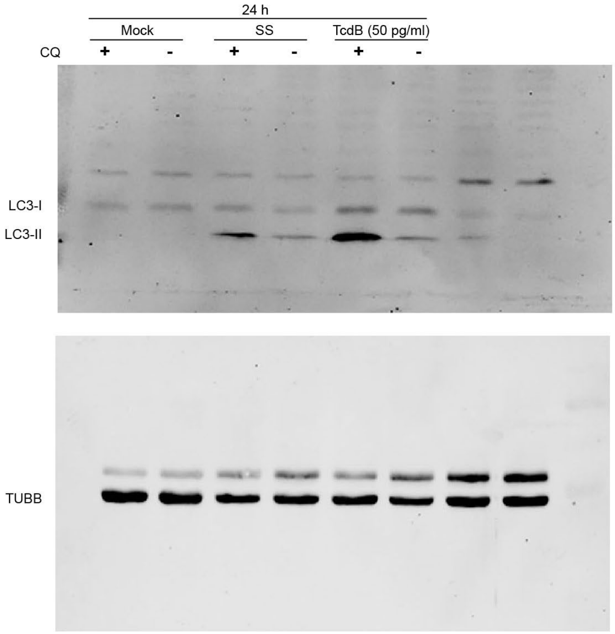

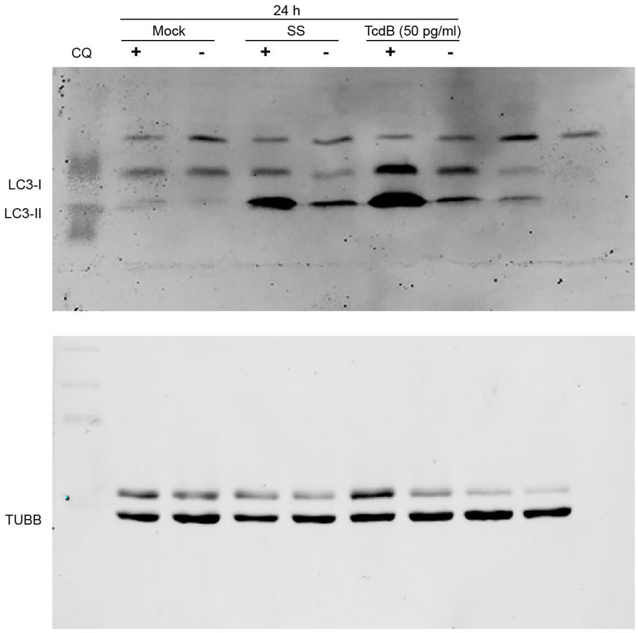

Figure S11. Assay of TcdB-triggered autophagy flux with or without CQ treatment. (Full length blot of Figure 1I)

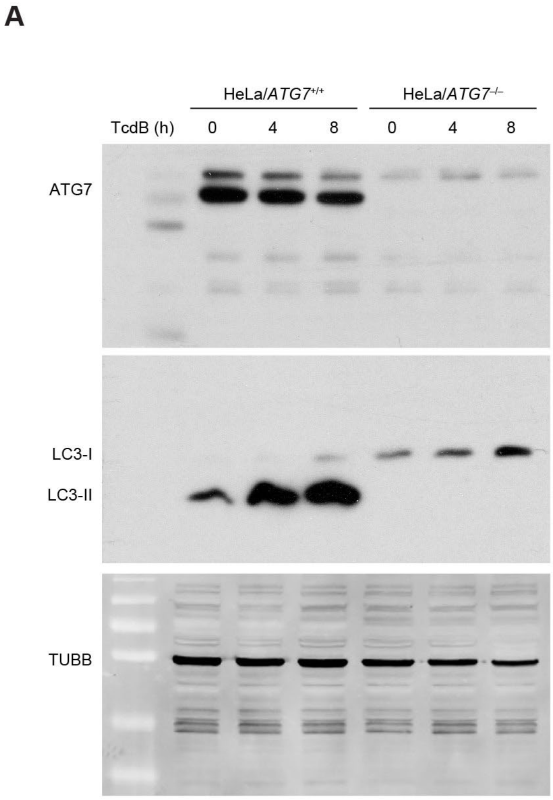

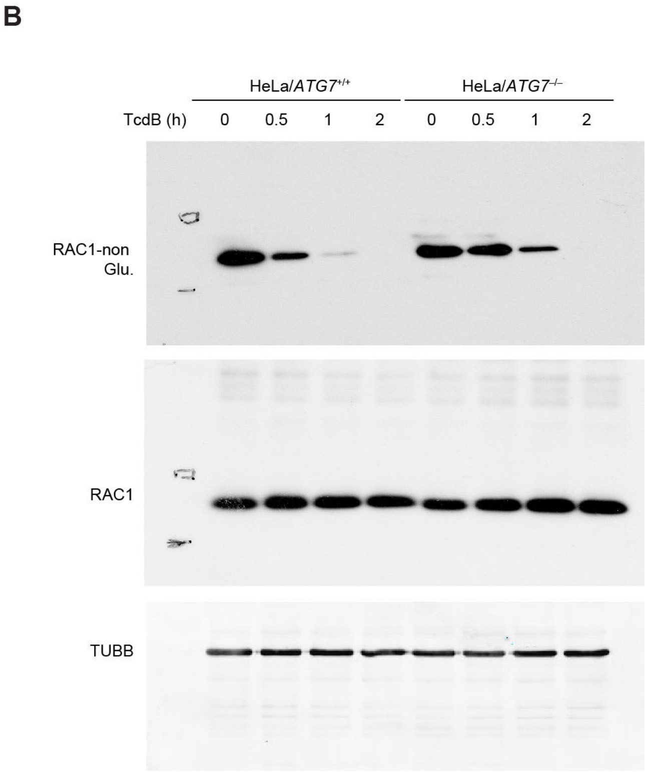

Figure S12. Effect of ATG7 deficiency on TcdB-induced autophagy TcdB-induced Rac1 glucosylation in HeLa cells. (Full length blot of Figure 2A and B)

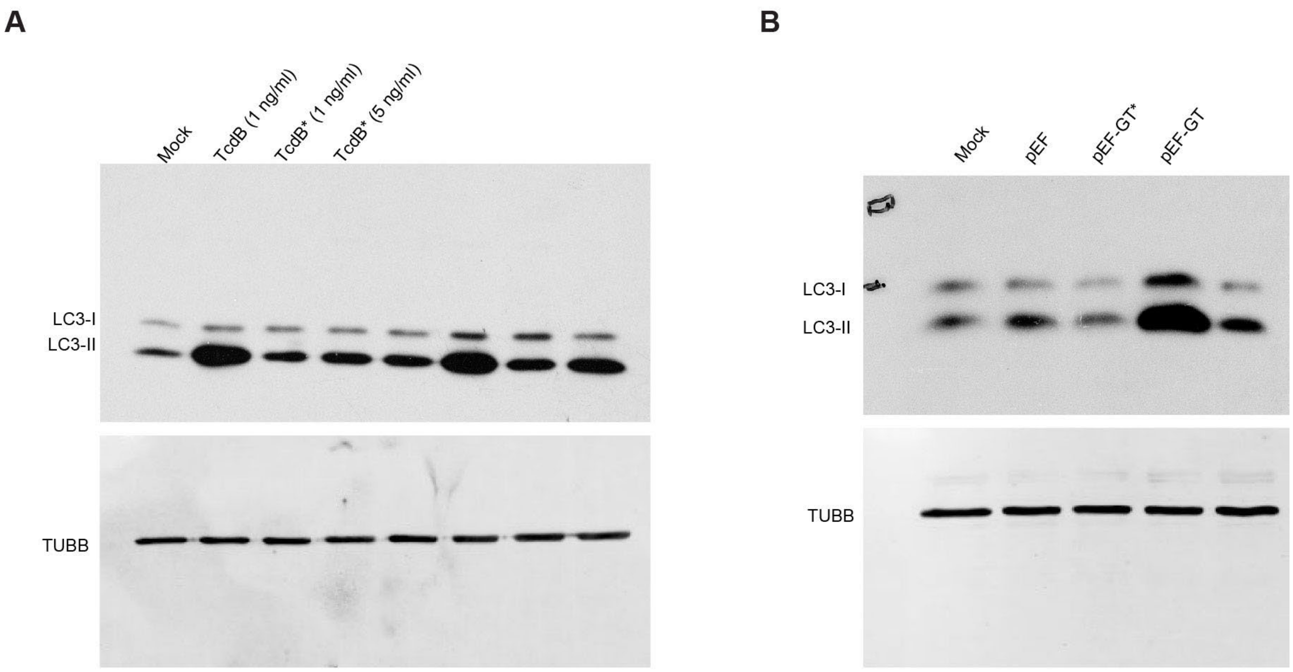

Figure S13. Effect of glucosyltransferase activity on TcdB-induced autophagy. (Full length blot of Figure 3B and E)

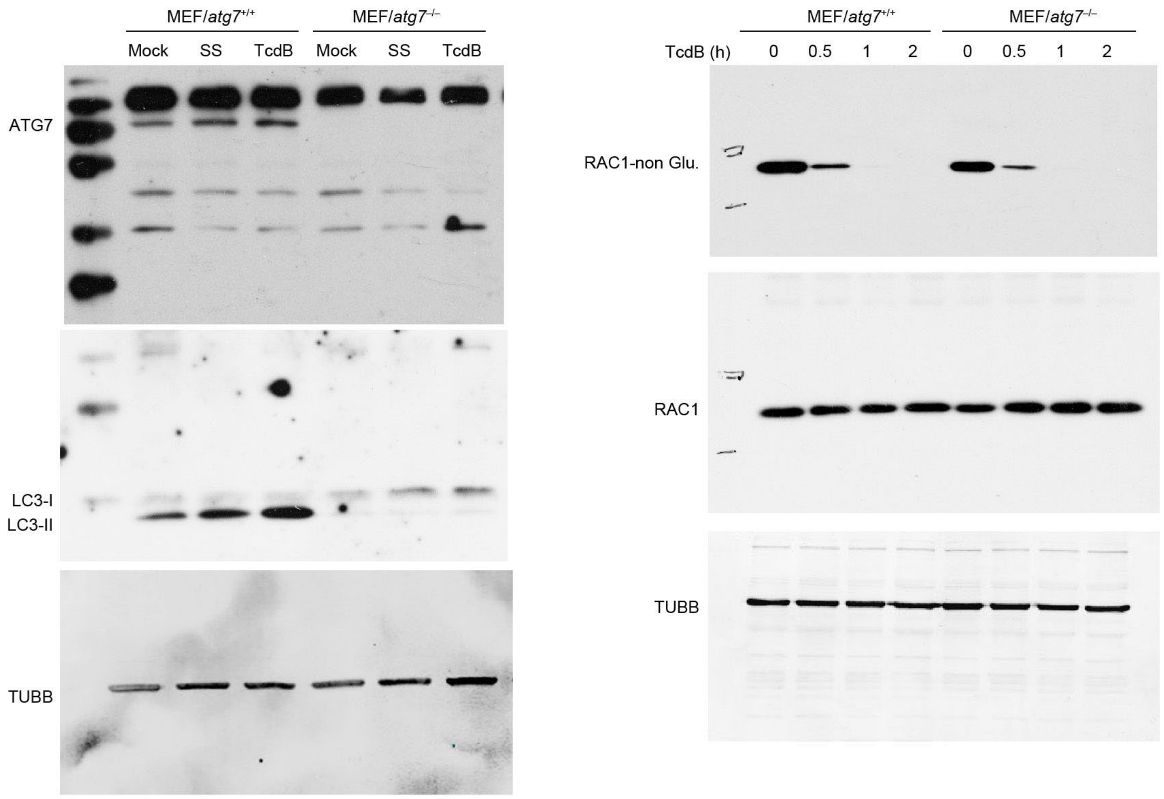

Figure S14. Effect of ATG7 deficiency on TcdB-induced autophagy (left) and Rac1 glucosylation (right) in MEF cells. (Full length blot of Figure 4E)

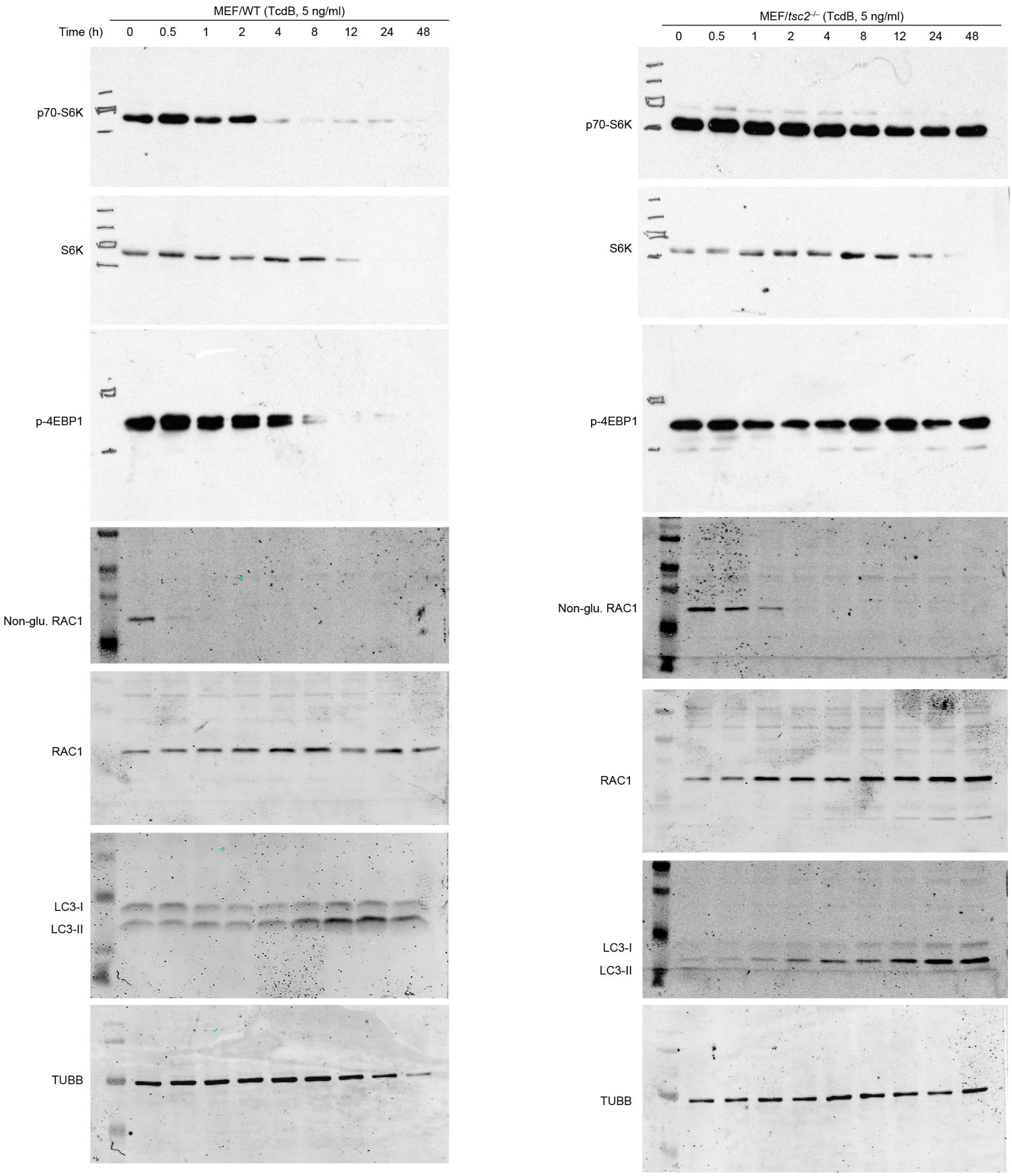

Figure S15. Immunoblotting analysis for the effect of TcdB on wild type (left) and tsc2-/-(right) MEFs.

(Full length blot of Figure 5A)

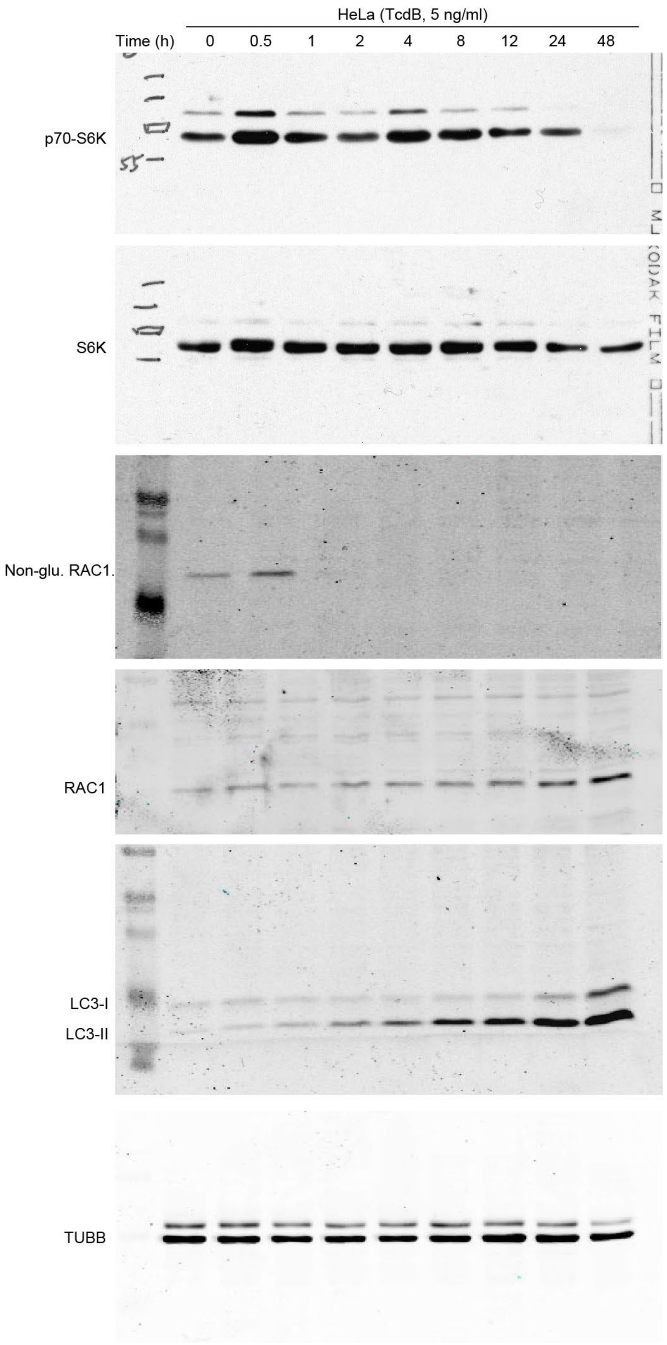

Figure S16. Immunoblotting analysis for the effect of TcdB (5 ng/ml) on HeLa cells. (Full length blot of Figure 5B)

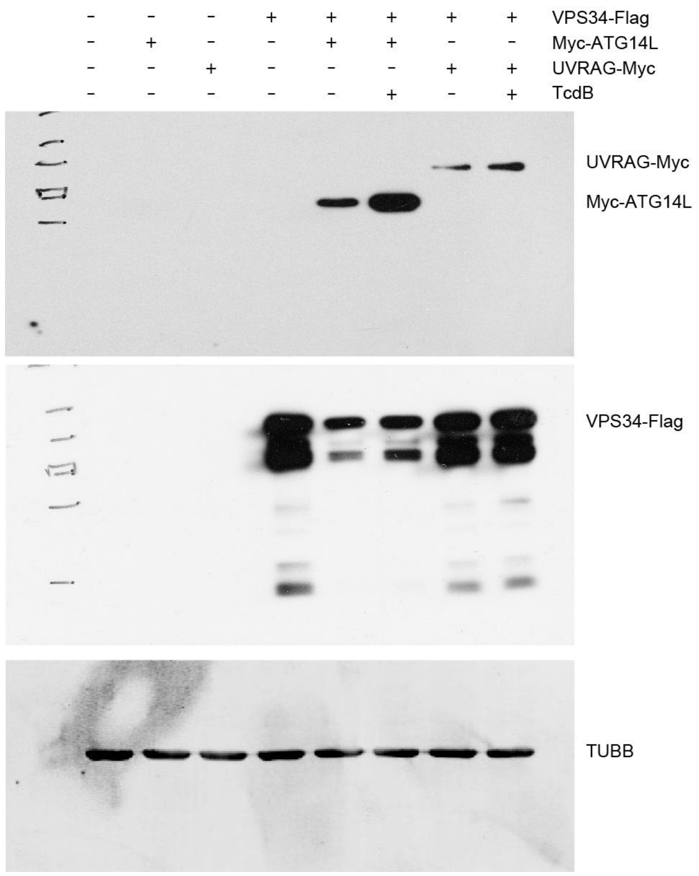

Figure S17. The protein-protein interaction between VPS34 and ATG14L or UVRAG with or without TcdB treatment. (Full length blot of Figure 6A)

Supplemental Table S1

List of Primers:

For pHis1522-TcdB & pHis1522-TcdB*	
rTcdB-F/BsrGI	5'-gcgcTGTACAATGAGTTTAGTTAATAGAAAACAGT-3'
rTcdB-R/SpeI	5'-ggACTAGTTGACATAAGAATAAAATCAT-3'
rTcdB-F/mutant1	5'-TTGGTGGTATGGCTTTAAATGTTAATATGTTA-3'
rTcdB-R/mutant1	5'-TAACATATTAACATTTAAAGCCATACCACCAA-3'
For pEF6-BSD-TcdB-GT & pEF6-BSD-TcdB-GT*	
rTcdB-F/BamHI	5'-cgGGATCCATGAGTTTAGTTAATAGAAAACAGT-3'
rTcdB-GT-R/EcoRI	5'-cgGAATTCTTATCATCTTCACCAAGAGAACCTTC-3'
rTcdB-F/mutant1	5'-TTGGTGGTATGGCTTTAAATGTTAATATGTTA-3'
rTcdB-R/mutant1	5'-TAACATATTAACATTTAAAGCCATACCACCAA-3'
For pCMV5-3 x Flag-Atg7	
3 x Flag-F1	5'-GGggtaccATGGACTACAAAGACCATGACGGTGATTATAAAGATCATGACATCGACTA-3'
3 x Flag-F2-Atg7	5'-TAAAGATCATGACATCGACTACAAGGATGACGATGACAAG ATGGCGGCAGCTACGGGG-3'
hAtg7-R/XbaI	5'-GCtctagaTCAGATGGTCTCATCATCGCTC-3'
For pEF6-BSD-Atg14L/UVRAG-myc-his-B	
hAtg14L-KpnI-F	5'-GGggtaccATGGCGTCTCCCAGTGGGAAG-3'
hAtg14L-EcoRI-R	5'-CGgaattcGAACGGTGTCCAGTGTAAGCT-3'
hUVRAG-KpnI-F	5'-GGggtaccATGAGCGCCTCCGCGTCG-3'
hUVRAG-SpeI-R	5'-GactagtACACTTATCGGAACTCCTGCG-3'
